# Risk factors for malaria infection prevalence and household vector density between mass distribution campaigns of long-lasting insecticidal nets in North-western Tanzania

**DOI:** 10.1186/s12936-020-03369-4

**Published:** 2020-08-20

**Authors:** Jacklin F. Mosha, Eliud Lukole, J. Derek Charlwood, Alexandra Wright, Mark Rowland, Olivia Bullock, Alphaxard Manjurano, William Kisinza, Franklin W. Mosha, Immo Kleinschmidt, Natacha Protopopoff

**Affiliations:** 1 National Institute for Medical Research, Mwanza Medical Research Centre, Mwanza, Tanzania; 2 Department of Disease Control, London School of Hygiene and Tropical Medicine, London, UK; 3 Kilimanjaro Christian Medical University College, Moshi, Tanzania; 4 Amani Medical Research Centre, National Institute for Medical Research, Muheza, Tanzania; 5 MRC Tropical Epidemiology Group, Department of Infectious Disease Epidemiology, London School of Hygiene and Tropical Medicine, London, UK

**Keywords:** Malaria, Llins, Effectiveness, Vectors, Tanzania

## Abstract

**Background:**

Long-lasting insecticidal nets (LLINs) are the most widely deployed vector control intervention in sub-Saharan Africa to prevent malaria. Recent reports indicate selection of pyrethroid insecticide resistance is widespread in mosquito vectors. This paper explores risk factors associated with malaria infection prevalence and vector density between mass distribution campaigns, changes in net coverage, and loss of protection in an area of high pyrethroid resistance in Northwest Tanzania.

**Methods:**

A cross sectional malaria survey of 3456 children was undertaken in 2014 in Muleba district, Kagera region west of Lake Victoria. Vector density was assessed using indoor light traps and outdoor tent traps. Anophelines were identified to species using PCR and tested for *Plasmodium falciparum* circumsporozoite protein. Logistic regression was used to identify household and environmental factors associated with malaria infection and regression binomial negative for vector density.

**Results:**

LLIN use was 27.7%. Only 16.9% of households had sufficient nets to cover all sleeping places. Malaria infection was independently associated with access to LLINs (OR: 0.57; 95% CI 0.34–0.98). LLINs less than 2 years old were slightly more protective than older LLINs (53 vs 65% prevalence of infection); however, there was no evidence that LLINs in good condition (hole index < 65) were more protective than LLINs, which were more holed. Other risk factors for malaria infection were age, group, altitude and house construction quality. Independent risk factors for vector density were consistent with malaria outcomes and included altitude, wind, livestock, house quality, open eaves and LLIN usage. Indoor collections comprised 4.6% *Anopheles funestus* and 95.4% *Anopheles gambiae* of which 4.5% were *Anopheles arabiensis* and 93.5% were *Anopheles gambiae *sensu stricto*.*

**Conclusion:**

Three years after the mass distribution campaign and despite top-ups, LLIN usage had declined considerably. While children living in households with access to LLINs were at lower risk of malaria, infection prevalence remained high even among users of LLINs in good condition. While effort should be made to maintain high coverage between campaigns, distribution of standard pyrethroid-only LLINs appears insufficient to prevent malaria transmission in this area of intense pyrethroid resistance.

## Background

Long-lasting insecticidal nets (LLINs) and indoor residual spraying (IRS) are the main vector control interventions deployed in sub-Saharan Africa to control malaria [1]. According to the World Health Organization (WHO), 69% of the estimated 663 million malaria cases averted during the 15 years after the millennium were attributed to the use of LLINs. Despite the increase in LLIN access, in recent years malaria has increased in several sub-Saharan countries [2]. A similar trend has emerged in Tanzania with malaria prevalence in children under 5 years old decreasing from 18% in 2007 to 9% in 2011 and then increasing to 14% in 2015 [3].

Insecticide-treated nets (ITNs) that required annual re-treatment were first distributed to pregnant women and infants through antenatal clinics in 2004. In 2008, LLINs that did not require re-treatment were distributed through mass campaigns targeting children under five years of age [4]. From 2011 onwards, distribution of LLINs was scaled up to cover the entire population, including adults, not previously reached. Approximately 18 million LLINs were distributed during this universal coverage campaign resulting in a net ownership (households owning at least one net) reaching 92% and usage of 72% [5].

ITNs are the most effective when usage is high to provide community protection [6]. The challenge, therefore, is to maintain high coverage between the mass distribution that occurs every 3–4 years [7]. Between universal coverage campaigns, LLIN coverage may be sustained by routine distributions through school and health facilities services [8, 9]. The insecticidal effectiveness of WHO recommended LLINs are shown to last for at least three years of use. However, the physical durability of the textile may be shorter than 3 years depending on usage practices and wear-and-tear. LLINs that are still insecticidal may be discarded due to accumulation of holes long before the end of the net’s insecticidal life [10].

The gains arising from increased usage of LLINs might also be undermined by the development of insecticide resistance among malaria vectors [11]. Resistance to pyrethroids is spreading across Africa, and has been reported in various districts of Tanzania [12]. While LLINs provided long term protection when mosquito vector populations in Tanzania were fully susceptible, effectiveness may become compromised in areas with high levels of pyrethroid resistance once LLINs deteriorate and develop holes [13, 14].

This study explored LLIN coverage indicators three years after the universal coverage campaign of 2011 and whether standard pyrethroid-only LLINs still provide protection against malaria vectors and infection prevalence in an area of intense pyrethroid resistance in Northwest Tanzania. It also explored other household and individual risk factors for malaria infection and vector density.

## Methods

### Study area

Cross sectional prevalence and entomological surveys were conducted during baseline of a cluster randomized controlled trial (RCT) assessing PBO LLINs and IRS with Actellic CS® (pirimiphos methyl) [15]. The study was conducted in 40 villages, divided in 48 clusters for the purpose of the RCT, in Muleba district (1° 45′ S 31° 40′ E), Kagera Region, North West Tanzania [15]. The study area covers 944 km^2^, 29,000 households and an estimated population of 135,000 and is situated between 1100 and 1600 m above sea level [15]. There are two rainy seasons: in October-December with an average monthly rainfall of 160 mm and in March–May with an average monthly rainfall of 300 mm [16]. Malaria transmission occurs throughout the year and peaks after the rainy seasons. Malaria prevalence infection in children under 15 years of age in the study area was 37% in July 2011, higher than the overall prevalence of 23% in the district during the same period [17].

*Anopheles gambiae *sensu stricto (*s.s*.) resistant to pyrethroids and *Anopheles arabiensis* were the only vectors found in 2011 [18]. Resistance testing in the area showed mortality against pyrethroid of 8.8% for *Anopheles gambiae* and 54.5% for *Anopheles funestus* and a resistance intensity of 38 fold for *Anopheles gambiae *sensu lato (*s.l.*) and 34 fold for *Anopheles funestus* compared to a susceptible mosquito strain [15].

Permethrin-treated LLINs (Olyset Net) were distributed through universal coverage campaign in 2011. After the campaign in 2011, net ownership was 90.8% and net usage was 55.7% [16]. Bendiocarb IRS was sprayed in 2011 and 2013, with house coverage over 90% in 2011 [19]. In the years following the 2011 campaign, additional LLINs were distributed though government clinics to protect pregnant women and children under 5 years of age.

### Data collection

#### Cross sectional malaria parasitaemia and household surveys

The prevalence survey was carried out from September to October 2014 [15]. All households in the study area were mapped using hand-held Global Positioning System (GPS) units (Garmin Legend e-trex) and Expert GPS v3.8 (TopoGrafix) software. A total of 2880 households (60 households per cluster) were randomly selected amongst the census list to participate in a household survey. Up to three children aged 6 months to 14 years per house were randomly selected.

On obtaining household consent, a questionnaire was administered and data entered into a personal digital assistant (PDA, HP IPAQ 114 Classic Handheld) on number of residents, socio economic status, house construction and quality, education, access to and use of LLINs and other malaria preventive measures, and presence of livestock.

Selected children were given a coupon and asked to present the following day for parasitological testing. *Plasmodium* infection was assessed by a rapid diagnostic test (RDT) (CareStart RDTs; HRP2/pLDH, (pf/PAN), Diasys, Wokingham, UK) and haemoglobin levels measured using HemoCue Hb 201 + (Aktiebolaget Leo Diagnostics, USA). Results were initially recorded on paper forms and double entered into a digital database and checked for consistency.

In the same households selected for cross sectional survey, 20% of LLINs were inspected for assessment of physical condition and hole index determined according to WHO guidelines [20]. Nets were draped over a frame and holes counted, classified into categories of: less than 2 cm (size 1); 2–10 cm (size 2); > 10–25 cm (size 3) and > 25 cm (size 4), and proportional hole index (pHI) as an estimate of hole area was calculated. For purposes of analysis the LLINs were further categorized into three groups: good condition (pHI 0–64), moderately damaged (pHI 65–642) and badly damaged (pHI > 643) [21].

### Entomological monitoring

From November 2014 to January 2015, indoor mosquito collections were conducted using CDC light traps in 14–21 randomly selected houses per cluster for one night only. The light traps were installed at the foot of a bed occupied by a family member sleeping under a LLIN. In addition, two or three furvela tent trap collections per cluster [22] were conducted for one night to assess outdoor density. They were placed near to houses selected for indoor light trap collections. Further information on house structure, livestock, and LLINs access and usage was collected. All mosquitoes collected were morphologically identified to species [23]. Up to 20 *Anopheline* mosquitoes per house were subsequently tested for *Plasmodium falciparum* circumsporozoite protein (Pf-CSP) using ELISA [24]. A sub-sample of *An. gambiae s.l.* was tested using real time PCR Taq Man assay to distinguish between the two sibling species *An. gambiae s.s.* and *An. arabiensis* [25]. Species composition of *Anopheles funestus* complex was determined using conventional PCR [26].

### Statistical analysis

Statistical analysis was performed using STATA (version 12, College Station, TX, USA). Random effects logistic regression models were used to determine possible risk factors for malaria infection determined by RDT in children 0.5–14 years old adjusting for correlation of malaria infection within clusters.

Potential household and personal risk factors were explored: age, gender, individual net use, education of household head, social economic status (SES), quality of house construction and presence of open eaves, number of people per sleeping room (a proxy for crowding), LLIN ownership and access, indoor residual spraying (IRS) in 2013, presence and proximity of livestock.

Variables were cleaned and coded and those which were nonlinearly related to the outcome were categorized. Variables were created for improved housing structure, individual net use, household net access, household crowding and social economic status, detail of all variables is indicated in Table 1.

**Table 1:** Definition of variables

Indicators	Description
Age	Age was categorized into three groups: 0.5–1 year, 2–4 years and 5–14 years
Altitude	Altitude (metres) was categorized into three groups with the following cutoff Low altitude ranging (1128–1264), Medium altitude ranging (1265–1352) High altitude ranging (1353–1654)
Social economic status	SES was created as a weighted sum of data on household possessions and utilities, using principal components analysis and divided into tertiles
Improved housing structure	Is a binary variable where houses are coded as ‘yes’ if they have at least one of the following intact ceiling, brick walls, plastered walls, full cement floors and iron roofs
Household crowding	Number of people sleeping per room. Number of sleepers per room was dichotomized into groups with 1, and > 1 person sleeping in the room
Eaves of the house	Binary variable ‘open’ if household has open eaves or ‘closed’ if household has no open eaves
LLINs’ ownership	Binary variable ‘yes’ if household owns at least one LLIN
Individual LLIN condition	Physical condition of the net used by sleeper. The variable is categorized in. 1/ good condition net (PHI 0–64). 2/ moderate condition net (PHI 65–642) and 3/ torn net (PHI > 643)
Individual LLIN usage	This is a binary variable indicating whether individual is sleeping under a net or not
Household net access	Binary variable ‘yes’ if household owns enough net to cover every sleeping place. Which is number of LLINs owned (regardless if used or not) divided by the number of sleeping places
Indoor residual spraying (IRS)	Binary variable ‘yes’ if household was sprayed in 2013
Livestock present	Binary variable ‘yes’ if livestock are owned
Animal kept inside the house	Binary variable ‘yes’ if animals are sleeping inside the household, this included goats, sheep and cows
Level of education of head of household	Binary variable ‘yes’ if the head of household has at least completed primary education
Moderate to severe Anaemia	Defined as haemoglobin < 8 g/dL

All variables were analysed individually for an association with malaria infection (outcome) using a logistic regression that included village and household as random effects to account for correlation within individual village clusters and households. All potential risk factors showing evidence for a possible association with malaria infection (p < 0.1) were included in the preliminary main effect multivariate logistic regression model. A forward stepwise method was used, retaining any risk factors showing an association (LRT test p < 0.05). No a priori interaction terms were identified. Multi-collinearity was assessed for by removing parameters one by one from the model and examining the standard errors of the remaining parameters. If this caused the standard error of one of the remaining parameters to change by > 10% this was defined as multi-collinearity and one of the parameters removed.

A similar approach was adopted to assess risk factors for indoor vector density. Because vector density count data was skewed and often over dispersed, a negative binomial regression analysis was used. The model estimated a density rate ratio (DRR) as the measure of effect. Univariate regression of each risk factor was performed initially and the forward step-wise method was used to build a multivariate model.

## Results

### Household and study participants

**Fig. 1:**
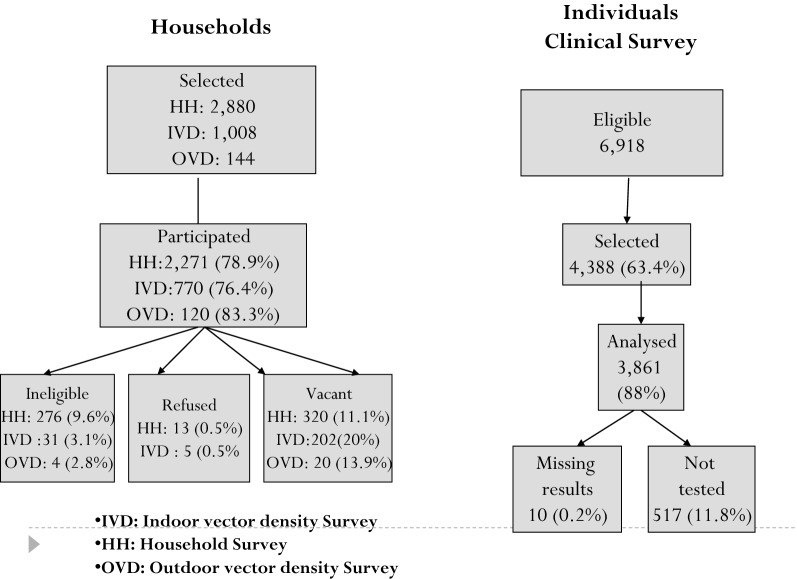
Study profile for household, individual selection and entomological monitoring

The total number of households which were selected to participate in the baseline survey was 2880. Of these 2270 (78.8%) consented to participate, 276 (9.6%) failed to meet the inclusion criteria (no children < 15 years), 13 (0.5%) refused and 11.1% were excluded for other reasons (Fig. 1). Of the 6918 children aged between 0.5 and 14 years, 4388 (63.4%) were selected for inclusion and of these 3871 participated in the clinical survey, 10 children had missing RDT results and 517 (11.8%) children did not turn up for testing. Therefore, of the total children selected 3861 (88%) were included in the analysis (Fig. 1).

Almost all nets which were used were LLINs (96.8%). The proportion of individuals who reported using a net the night before was 27.1%. The proportion of nets declared to be less than 2 years old was 37.1%, those 2–3 years old was 31.3% and those over 3 years old were 31.4%. Many of the nets had been received via clinic-based distribution during the period since the last UCC. Only 26% of individuals slept under nets of good condition, 35% slept under nets of moderate condition and 38% under nets which were badly torn. Net access was very low; only 16.9% of individuals were residing in households with enough nets to cover all sleeping places. About 25.9% of the households reported to be sprayed with insecticide in the year before (Table 2).

**Table 2:** Factors associated with malaria infection in the univariate and multivariate analysis

	Number of children	% with malaria infection (%)	Univariate			Multivariate		
			OR	96% CI	p-value	OR	96% CI	p-value
Altitude (metres)								
Low (1128–1264)	1300	74.7	1					
Medium (1265–1352)	1291	68.1	0.38	0.28–0.50	0.001	0.78	0.42–1.43	0.416
High (1353–1654)	1266	51.0	0.14	0.10–0.20	0.001	0.27	0.14–0.55	< 0.001
Age								
< 1 year	384	50.5	1					
2–4 years	1062	63.0	1.86	1.44–2.41	< 0.001	2.59	1.31–5.11	0.006
5–14 years	2414	67.7	2.52	1.99–3.20	< 0.001	3.82	1.89–7.72	< 0.001
Improved housing structure								
No	589	76.9	1			1		
Yes	3272	62.5	0.32	0.22–0.48	< 0.001	0.27	0.13–0.54	< 0.001
Socio economic status								
Poorest	1261	72.6	1			1		
Middle	1264	64.3	0.72	0.59–0.86	0.001	0.98	0.51–1.89	0.961
Least poor	1258	57.6	0.50	0.42–0.60	< 0.001	1.07	0.52–2.19	0.862
Adequate access to LLINs								
No	3167	66.0	1			1		
Yes	643	58.8	0.68	0.57–0.83	< 0.001	0.57	0.34–0.98	0.042
Individual net age								
< 2 years	350	52.9	1			1		
2–3 years	295	64.1	2.32	1.26–4.28	0.007	1.89	1.01–3.53	0.044
4 years and more	299	65.9	2.37	1.28–4.37	0.006	1.90	1.02–3.58	0.004
Sex								
Male	1869	66.0	1					
Female	1870	63.4	0.87	0.75–1.01	0.061			
Eaves of the house								
Open	2334	69.2	1					
Closed	1478	57.9	0.59	0.51–0.69	< 0.001			
Livestock present								
No	1026	67.6	1					
Yes	2786	63.7	0.77	0.65–0.91	0.002			
Animals kept inside the house								
No	1857	63.5	1					
Yes	935	63.6	0.97	0.81–1.17	0.757			
Head of household attended school								
No	1007	71.4	1					
Yes (any schooling)	2791	62.4	0.74	0.62–0.88	0.001			
IRS 18 months before								
No	2798	67.0	1					
Yes	979	59.1	0.84	0.70–1.01	0.06			
Household owns at least 1 net								
No	1165	69.1	1					
Yes	2647	62.6	0.71	0.60–0.83	< 0.001			
Individual usage of LLIN								
Don’t use net	2276	66.4	1					
Use net	1045	61.2	0.69	0.50–0.94	0.018			
Individual LLIN condition								
Use good condition net (PHI > 64)	154	68.2	1					
Use moderate condition net (PHI 65–642)	206	61.7	0.65	0.28–1.49	0.307			
Use torn net (PHI > 643)	221	63.8	0.77	0.34–1.77	0.544			

Of the 1008 households selected for indoor entomological collection 770 participated, and of 144 selected for outdoor collection 120 participated (Fig. 1). Altitude of study households ranged from 1128 to 1654 m above sea level. Around 76.8% of households were constructed with mud walls and floors, and 61.2% of houses had open eaves. Most households (94.3%) relied on farming or fishing and selling cash crops for income. Most individuals came from houses with livestock (73.1%) and 33.5% of these kept animals inside the household. 73.3% of heads of households had attended primary school (Table 3).

**Table 3:** Risk factors associated with vector density in the univariate and multivariate models

	Number of HH	Indoor vector density	Univariate			Multivariate		
			IRR	96% CI	p-value	IRR	96% CI	p-value
Altitude (metres)								
1133–1242	192	63.2	1			1		
1243–1302	191	22.6	0.63	0.49–0.80	< 0.001	0.58	0.46–0.74	< 0.001
1303–1373	192	6.8	0.51	0.40–0.66	< 0.001	0.44	0.33–0.58	< 0.001
1374–1651	191	5.7	0.39	0.29–0.51	< 0.001	0.35	0.27–0.46	< 0.001
Wind during collection								
None/light	433	28.2	1			1		
Moderate/strong	335	19.9	0.77	0.64–0.93	0.007	0.69	0.57–0.83	< 0.001
Livestock								
No	267	17.5	1			1		
Yes	502	28.3	1.13	0.95–1.35	0.159	1.35	1.12–1.62	0.001
Improved housing structure								
No	579	29.3	1			1		
Yes	183	10.1	0.78	0.63–0.95	0.016	0.83	0.67–1.02	0.072
Household using at least 1 LLIN								
No	264	22.9	1			1		
Yes	505	25.4	0.79	0.66–0.93	0.006	0.78	0.66–0.92	0.004
Number of rooms	770	24.9	0.89	0.84–095	< 0.001	0.90	0.84–0.96	0.001
Household cooks inside the house								
No	620	23.3	1					
Yes	149	29.6	1.31	1.07–1.59	0.010			
Total time house has been sprayed	756	24.9	0.94	0.90–0.99	0.011			
Animal inside the house								
No	308	29.2	1					
Yes	196	26.5	1.01	0.82–1.25	0.933			
Rain during collection								
None	435	23.7	1					
Light	164	37.1	0.98	0.78–1.24	0.894			
Heavy	169	14.5	1.15	0.92–1.44	0.234			
Eaves of the house								
Closed	324	22.9	1					
Open	445	25.7	1.17	0.99–1.38	0.071			

### *Plasmodium* infection prevalence and risk factors for malaria infection

Overall malaria infection prevalence (all *Plasmodium* species*)* was 64.8% (95% CI 61.8–67.8) in children aged 6 months to 14 years old. The prevalence of clinical malaria, defined as fever (ear temperature, ≥ 37.5 °C) and any *Plasmodium* infection by RDT was 6.8% (95% CI 5.5–8.1). Only 2.9% (114) of tested children had fever and among those who had fever 86% had a positive test result. The prevalence of moderate or severe anaemia (haemoglobin < 8 g/dL—see Table 1 for variables’ definitions) in children under five was 19.5% (95% CI 17.9–21.4). Among anaemic children 88.9% (95% CI 85.5–92.2) had malaria infection. The odds of moderate-severe anaemia was six times higher if the child had malaria infection (OR 6.2, 95% CI 5.0–7.6).

In the univariate analysis the odds of malaria infection was lower with living at higher altitudes, in children in younger age groups, with better education, improved house structure, closed eaves, greater wealth, presence of livestock, net ownership, adequate access to LLINs, use of LLINs and with newer LLINs (Table 2).

In the multivariate model, malaria infection was independently associated with altitude, age, quality of housing structure, and adequate net access per sleeping place. The odds of malaria infection decreased with increasing altitude. Individuals living at high altitude had much lower odds of malaria infection compared to those who were living at lower altitude (OR 0.18 95% CI 0.13–0.26).

The odds of malaria infection increased with age, the oldest group being the most at risk (OR 3.82, 95% CI 1.89–7.72). Children living in improved housing structure had lower odds of malaria infection compared to those who were living in unimproved housing (OR 0.27, 95% CI 0.13–0.54). Individuals living in houses with an open eaves showed a strong association with malaria infection compared to those who were living in houses with closed eave gaps (OR 0.59; 95% CI 0.51–0.69).

Individuals who were living in households with adequate net access per sleeping place were better protected from malaria infection (59 vs 66%, OR 0.57; 95% CI 0.34–0.98, p = 0.042). Age of LLINs was also significant, with only LLINs less than two years of age showing evidence of protection (53 vs 65%, p = 0.004). LLINs’ usage was not associated with malaria prevalence in the multivariate analysis. There was no association between malaria protection and the hole index of LLINs over the WHO LLIN categories of good condition (32% infection prevalence, 49/154) to ‘unserviceable’ condition (36%, 80/221).

None of the other factors (gender, wealth, livestock, household head education, IRS spraying 18 months earlier, household owning at least one LLIN, and usage of LLINs of different physical conditions) were associated with malaria infection once adjusted for the other factors in the model.

### Mosquito fauna and risk factors for vector density

A total of 29,401 and 2668 mosquitoes were collected in 770 indoor light trap collections and in 120 outdoor tent trap collections respectively. Anophelines comprised 64% of the indoor collections and 57% of the outdoor collections. Mean vector density per collection night was 24.5 indoors and 12.7 outdoors. Of the Anophelines collected 4.6% were identified as *An. funestus s.l.* and the remaining were *An. gambiae s.l..* The 969 *An. gambiae s.l.* were identified to species by PCR, 93.5% were *An. gambiae s.s.*, 4.5% were *An. arabiensis*, and 2.2% did not amplify*.* There was no evidence of difference in ratio of *An. gambiae s.s.* to *An. arabiensis* between indoor and outdoor collections (X^2^ 3.5, p = 0.18). Of the 289 *An. funestus* identified by PCR 81.7% were *An. funestus s.s.,* 15.6% *Anopheles leesoni,* 1.0% *Anopheles parensis* and 1.7% did not amplify*.* A subsample of 4311 specimens was tested for CSP. The sporozoite rate was 4.7%. Both *An. funestus* and *An. gambiae s.s.* were found positive for CSP. The entomological inoculation rate was 0.34 (95% CI 0.20–0.49) infectious bites per person-night during the period of collection, and this rate varied from 0 to 2.7 between village clusters.

Altitude of households selected during the entomology survey ranged from 1133 to 1651 m, with a mean of 1303 m. It rained during 43.4% of collections and the wind was moderate or strong during 43.6% of collections. 34.7% of households did not have animals. For those that owned animals, 39% kept them indoor. 50% of families had poorly constructed houses and 24% had improved houses. 57.9% of houses had open eaves. The median number of rooms was 5 per house and median household size was 5 persons. Only 19.4% of families cooked inside the house. Houses had been sprayed 3 times since the first IRS campaign in 2007. An average of 65.7% of households were using at least one LLINs.

The univariate analyses showed evidence for negative associations between vector density and improved housing, number of rooms, households using at least one LLINs and number of times the house had been sprayed. Cooking inside the house was associated with higher vector densities. Altitude above 1242 m was associated with a decreased risk of indoor vectors, whereas light wind or no wind during collection was associated with increased risk.

House structure had an IRR of 0.75 (95% CI 0.60–0.90), indicating a 25% decreased risk of vector exposure in improved housing. Using at least one LLIN in the household and several times sprayed was associated with reduced vector density. There was no evidence of association with rain during collection night, open eaves or animals present inside the house.

The multivariate model retained five risk factors as significant: altitude, wind during collection, presence of livestock, house construction and LLINs’ usage. Number of rooms, showed co-linearity with house construction and, was therefore removed from the model. There was strong association with vector density, altitude and wind. Vector density was negatively associated with increasing altitude, being 65% less at the higher altitudes (above 1353 m) and with nights with moderate or strong winds. Improved housing structure was associated with reduced vector density whereas presence of livestock was associated with higher vector density. Net usage was associated with density after adjusting for other factors. The variables that showed no association with vector density in the multivariate model were: history of IRS, cooking indoors and presence of open eaves.

## Discussion

This study investigated the effects of LLINs three years after the last universal coverage campaign of 2011 in an area of intense pyrethroid resistance in Northwest Tanzania. The aim of the study was to explore the effects of net coverage and deterioration as well as other household and individual risk factors associated to malaria infection and vector density. At the time of the study many of the original LLINs had developed holes or been replaced by newer ones. Malaria prevalence was over 60% in the study area in 2014, while the estimated number of infective bites per day was 0.34. These rates were much higher than the rates observed in the same area in 2011 shortly after the campaign [17]. An increase in malaria infection was also observed in other regions during the same period. After the initial decrease in malaria infection to 9% (ranging 1–25%), malaria prevalence increased to 14% (ranging from 1 to 41%) across seven regions of the country by 2014 [3]. Various factors may have contributed to the rebound of malaria in the study area: these include reduced LLIN effectiveness caused by increasing insecticide resistance combined with deterioration of the LLINs’ physical condition, and reduced coverage and usage of LLINs.

LLIN ownership had decreased from 90.8% after the universal coverage campaign of 2011 to 69% in 2014 [16] and usage from 55.7 to 27.7% during the same period. While good household access to LLINs and self-reported individual LLIN use were both associated with reduced risk of malaria infection in the risk factor analysis, the reduction in LLIN coverage and usage in the years after the campaign would have contributed to the observed increase in malaria prevalence in 2014. The reported reasons for not using LLINs included: LLINs becoming torn over time and the poor access to new LLINs, which is consistent with findings from other studies [27]. Even in households with adequate access to LLINs malaria infection prevalence was still 59%, indicating loss of effectiveness of LLINs due to other reasons.

In the present study, access to LLINs was associated with malaria protection in the multivariate analysis whereas usage was not. This is not the first community study showing that net use is not associated with malaria. Net use last night is based on self-reporting by household members while access and ownership is observed and may be more reliable net coverage indicators [28, 29].

About 53% of the nets which were still in use had holes and around 35% of the nets were badly torn (pHI > 643). Previous studies have indicated that personal and community effectiveness of LLINs declines over time due to factors such as net coverage, ageing of nets, net damage and insecticide resistance [13, 14]. Protection against highly resistant mosquitoes is reduced when LLINs develop holes because such mosquitoes are more able to penetrate the net via the holes, bite and blood feed [13, 14]. However, the level of protection was not associated with hole index category. It is possible the sample size was underpowered; only 581 (15%) of the 3861 children tested for malaria had their nets assessed for hole index. Nevertheless, even a small degree of holing might be sufficient for a LLIN to lose its capacity to protect when pyrethroid resistance reaches high frequency and intensity [13, 14]. There was significantly greater protection from malaria among users of LLINs less than two years of age; such nets would have fewer holes, higher concentrations of pyrethroid and were potentially more repellent to insecticide resistant mosquitoes. Once nets are more than two years of age they lose some capacity to protect against mosquitoes that are highly resistant. This observation should be kept in perspective; the actual difference in infection prevalence was between 53% in users of LLINs < 2 years old and 64% in LLINs > 2 years old. Standard LLINs were simply no longer adequately protective against this highly resistant vector population.

Other main risk factors for malaria infection prevalence in children 6 months to 14 years included living in area of lower altitude, in poorly constructed houses and with open eaves, being 5–14 years old, and coming from a lower socio-economic category. The same risk factors are also associated with higher vector density. These findings are consistent with other studies [30]. Some studies have reported an increased parasitaemia in children living at low altitudes compared to those who lived at high altitudes [31]. High altitudes are associated with lower temperatures, which have an impact on growth of mosquito larvae and therefore mosquito abundance [32]. Lower temperature increases the interval between biting/feeding cycles and also slows the rate of malaria parasite development within the mosquitoes.

The observed increased risk of malaria infection in people living in poor housing is consistent with previous studies [33, 34]. Living in improved housing was also associated with reduced vector density. A recent systematic review and meta-analysis found that the odds of malaria infection was 47% lower among residents of modern houses compared to traditional houses [34]. House designs with open eaves and unscreened windows have been associated with increased risk for malaria infection, as a gap in the eaves is an entry point for malaria vectors [35].

Increased risk of malaria infection in older children (the 5–14 year age group) compared to younger children seen in this study is a trend which has been observed in other studies conducted elsewhere in Tanzania [36]. Older children have higher exposure to mosquito bites compared to younger age groups as they may tend to sleep later and less likely to use LLINs in some studies and places [37]. In addition, due to the acquired anti-malarial immunity in older children, most have persistent asymptomatic malaria infections that are less likely to be treated, unlike younger children [38].

Households that own livestock had higher vector density, but this did not translate in increased malaria infection prevalence when adjusted for other co-variates. This suggests that livestock may attract *Anopheles,* but did not lead to an increase in human biting rate. The impact that livestock have on malaria and vector density is complex. Some studies indicate that livestock provides zooprophylatic protection [39], while other studies showed that livestock increases risk [40]. It depends on how the livestock are deployed within the household compound relative to humans and the anthrophilic/zoophilic plasticity of the vector species. Further study of vector composition between household with or without livestock would be required to better understand the difference in the present study.

The study has some limitations. The prevalence cross sectional survey was carried out before the entomological collections potentially affecting level of net coverage in the households and therefore comparability. However similar risk factors were associated with both outcomes showing that this one-month time lag may not have affected the findings of the study. Net usage assessment was based on self-reporting by household members and might not be reliable which could explain the lack of association between usage and malaria infection prevalence.

## Conclusion

Three years after the universal coverage campaign LLIN usage had decreased considerably despite distribution of new LLINs in the interim. Children living in households with full access to LLINs per sleeping place were at lower risk of malaria; however, malaria prevalence in this group remained high. Standard pyrethroid-only LLINs were no longer sufficiently protective even when in good condition and relatively new. More efforts should be made to maintain high coverage of more effective types of LLIN between campaigns.

## Data Availability

The datasets used and/or analysed during the current study are available from the corresponding author on reasonable request.
